# Bilateral ovarian teratoma complicated with carcinosarcoma in a 68 year old woman: a case report

**DOI:** 10.1186/1471-2407-11-218

**Published:** 2011-06-03

**Authors:** S Shanmughapriya, G SenthilKumar, K Balakrishnan, N Vasanthi, K Vinodhini, K Natarajaseenivasan

**Affiliations:** 1 Department of Biochemistry, School of Life Sciences, Bharathidasan University, Tiruchirappalli, Tamilnadu, India; 2 Department of Surgical Oncology, Dr. G. Vishwanathan Hospital, Tiruchirappalli Tamilnadu, India; 3 Department of Pathology, K.A.P.Viswanathan Govt Medical College, Tiruchirappalli Tamilnadu, India; 4 Medical Microbiology Laboratory, Department of Microbiology, School of Life Sciences, Bharathidasan University, Tiruchirappalli, Tamilnadu, India

## Abstract

**Background:**

Composing of less than 1% of all ovarian cancers, immature teratoma is a malignancy that mainly affects the young, and they present with advanced disease. The treatment of immature teratoma is conservative primary surgery usually involving unilateral salpingo-oophorectomy followed by combination chemotherapy.

**Case presentation:**

Here we present a case of a 68 year old woman with bilateral ovarian teratoma complicated with carcinosarcoma. The patient was diagnosed as FIGO stage IIIC. She underwent neoadjuvant chemotherapy and interval cytoreduction followed by optimal cytoreduction. The post operative management strategies and gynaecological follow up studies revealed no evidence of regional or distant metastasis.

**Conclusion:**

Thus the choice of initial treatment should be decided in a selective fashion depending on various prognostic factors in order to increase the survival of the patients.

## Background

The term teratoma was derived from the Greek root teratos which means Monster [1]. Teratomas are the most common germ cell tumours (GCTs) composing of two or more germ layers (ectoderm, mesoderm or endoderm), derived from a pluripotent malignant precursor cell. Mature teratomas consist of adult-type differentiated components such as cartilage and glandular epithelium and immature teratomas contain tissues with partial somatic differentiation similar to that in foetal tissues [2]. Composing of less than 1% of all ovarian cancers, immature teratoma is rapidly progressing without treatment. The average age at diagnosis of this non-dysgerminatous tumour is 19 years. The symptoms are often non-specific, usually consisting of mass effect inflicting abdominal/pelvic discomfort [3]. The foundations of treatment for immature teratoma have been steadfast throughout decades: conservative primary surgery usually involving unilateral salpingo-oophorectomy followed by combination chemotherapy. We present here a patient with bilateral ovarian teratoma complicated with carcinosarcoma at the age of 68. Women at this age group are less likely to be optimally debulked, more likely to have high rates of chemotherapeutic toxicity and high rate of medical co-morbidities [4,5]. Taking into account all these considerations the present study was formulated to determine the impact and success of neoadjuvant chemotherapy and interval cytoreduction in the treatment of ovarian teratoma in an old age woman.

## Case presentation

A 68 year old lady was referred to oncology outpatients in January 2010, with a month history of severe abdominal pain. Her past clinical history included no tubal ligation or hormone replacement therapy. She had previously given birth to a female child at her age of 21. The patient was fit and well with no significant past medical history apart from hypertension and diabetes. There was no family history of breast or ovarian carcinoma.

Physical examination revealed an abdominal pelvic mass with ascites and omental deposits. Blood analysis showed haemoglobin concentration of 11.3 g/dl while the rest of the analysis were normal including the carcinoembryonic antigen (CEA), alphafetoprotein and Cancer Antigen-125 (CA-125) (1.25 U/ml). The cytological examination of the ascitic fluid showed cellular smear composing of mixed inflammatory cells admixed with papillary and acinar clusters of eosinophilic cells with pleomorphic hyperchromatic nuclei thus suggesting a metastatic carcinoma.

**Figure 1:**
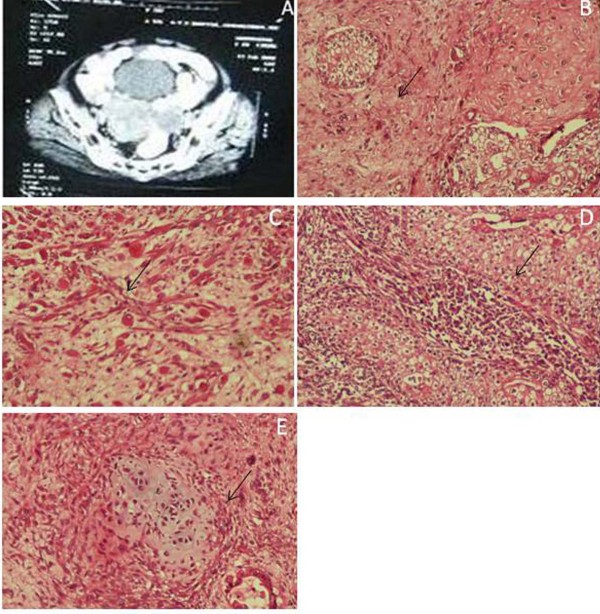
**Clinical observations of the malignant teratoma of the ovary**. A: Computerised tomography of the abdomen, B: Characteristics of carcinosarcoma, C: Stroma with rhabdoid elements, D: Malignant changes of squamous layer, E: Stroma with cartilaginous elements.

Pelvic sonogram revealed a large tumour mass with solid and cystic components. Minimal ascites was noted. A subsequent computerized tomography (CT) scan of the abdomen and pelvis revealed heterogeneously enhancing mass lesion measuring 9 × 7.1 cm with solid and cystic areas and calcification in the retrovesical region (Figure 1A). The mass was found to be compressing on the right lower ureter leading to right hydrodeuteronephrosis. Multiple enlarged peritoneal nodules with a largest one measuring 10 × 6.3 cm were observed. Moderate free fluid in abdomen and pelvis with moderate right pleural effusion was observed. The diagnosis of the malignant transformation was suggested by the invasive growth of soft tissue components through the teratoma wall by CT scan images. Finally based on the clinical manifestations she was diagnosed as having FIGO stage IIIC of immature teratoma.

Due to unresectable bulky tumours and poor performance status the patient underwent neoadjuvant chemotherapy (NAC) followed by Interval Cytoreductive Surgery (ICS). Four cycles of combination of paclitaxel and carboplatin were administered every 3 weeks. ICS was performed in the 5^th ^week after administration of the 4^th ^cycle of NAC. Standard procedures of ICS consisting of total abdominal hysterectomy, bilateral salpingo-oophorectomy, omentectomy and maximal debulking of metastatic tumour was adopted. Following the procedure there was no residual macroscopic disease and she was transferred to high dependency.

The surface of the tumour appeared rough and congested. On cutting, the ovarian mass was full of sebaceous material and hair densely adherent to surrounding structures. Histopathological examination confirmed bilateral teratoma complicated with carcinosarcoma (Figure 1B) with heterogeneous rhabdoid elements (Figure 1C) Microscopically the left ovarian tumour displayed variable size cyst lined by multilayered malignant squamous cells (Figure 1D) with rhabdoid spindle cells, cytoplasmic clearing, mature atypical cartilage (Figure 1E), malignant tubules, small round cells with rosettes, bone marrow and neural bundle. In addition the focal area showed atypical giant bizarre cells. The observation of the right ovarian tumour displayed admixture of malignant, epithelial and mesenchymal elements. The epithelial layers showed variable sized islands of squamoid and polygonal spindle cells and rarely showed tubular papillary structure. The stroma appeared to be a mixture of rhabdoid spindle cells, primitive mesenchymal cells, neural elements, adipocytic elements. Focal area showed pigmented cells.

The patient recovered well from surgery and was referred for oncological follow up and post- surgical chemotherapy (same regime as NAC). Given her age and performance status a surveillance approach was taken with regular clinical examinations, serial tumour markers and routine CT scans. The follow up studies showed no evidence of recurrence, regional or distant metastasis.

## Conclusion

The prognosis of immature teratoma heavily depends on the FIGO stage. The other prognostic factors include tumour grade, growth pattern, capsular rupture and vascular invasion [6,7]. Age alone has been shown to be an independent predictor of survival in those with ovarian cancer [8]. The 2-year disease free survival for grade 1, grade 2 and grade 3 is 83%, 50% and 33% respectively [9]. The general consensus in the treatment of stage I immature teratoma is unilateral salpingo-oophorectomy of the involved ovary for grade I disease, followed by adjuvant chemotherapy if tumour is grade 2 or 3 [10,11]. Several controversial arise regarding the standard treatment of immature teratoma among paediatric/adolescent population [12,13].

In spite of the controversial in treatment strategy, in the present study we have presented a rare event of bilateral ovarian teratoma complicated with carcinosarcoma in a 68 year old woman. The FIGO stage was IIIC. The rare event coupled with her age suggests that she was less likely to be treated with standard therapy [14-16] and she was less likely to tolerate these treatments when received [17]. Taking into account her age factor she was less likely to be optimally debulked and also necessitate a post operative intensive care stay [18].

Thus in a view point not to worsen the prognosis for patient with such advanced ovarian cancer, neoadjuvant chemotherapy followed by interval cytoreduction was suggested. Thus caring for older women with advanced ovarian cancers presents unique challenges. Even after the achievement of optimal cytoreduction the survival rates are found to be very low. However careful selection of primary cytoreduction versus neoadjuvant chemotherapy can result in higher rates of optimal cytoreduction, low rates of post-operative death. Thus in patients where optimal debulking is not possible at presentation, neoadjuvant chemotherapy will be the best choice of treatment.

## Consent

Written informed consent was obtained from the patient for publication of this case report and any accompanying images. A copy of the written consent is available for review by the Editor-in-Chief of this journal.

## Competing interests

The authors declare that they have no competing interests.

## Authors' contributions

SS and NSK designed the study and drafted the MS, SKG managed the treatment strategy, BK carried out the histopathological findings, VN and VK revised the manuscript critically. All authors have read and approved the final manuscript.

## Pre-publication history

The pre-publication history for this paper can be accessed here:

http://www.biomedcentral.com/1471-2407/11/218/prepub
